# PyBioPAX: biological pathway exchange in Python

**DOI:** 10.21105/joss.04136

**Published:** 2022-03-11

**Authors:** Benjamin M. Gyori, Charles Tapley Hoyt

**Affiliations:** 1 Laboratory of Systems Pharmacology, Harvard Medical School

## Statement of need

Understanding the complex molecular processes governing how cells respond to external stimuli crucially relies on prior knowledge about signaling, regulatory, and metabolic pathways. Standardized representations are necessary to exchange such pathway knowledge and allow interoperability between tools. BioPAX (Demir et al., 2010) is a widely used pathway exchange format that is formally defined in the BioPAX Language Specification. BioPAX is serialized into the Web Ontology Language (OWL) format, typically as RDF/XML. Software support for parsing, serializing, and finding patterns in BioPAX models is implemented in the Paxtools Java package (Demir et al., 2013). However, interacting with Paxtools is difficult from Python, and requires running a Java Virtual Machine via cross-language frameworks such as pyjnius (Kivy, 2021). Therefore, there is a need for native Python software support for BioPAX to facilitate integration with widely used systems biology tools (e.g., PySB (Lopez et al., 2013), Tellurium (Medley et al., 2018), PyBEL (Hoyt et al., 2018)), and pathway analysis workflows more generally.

### State of the field

Support for the BioPAX language is implemented in the Paxtools Java package (Demir et al., 2013) and a wrapper extension around it called PaxtoolsR enabling its usage from an R environment (Luna et al., 2016). A graphical tool for the visualization of BioPAX called ChiBE (Babur et al., 2010) is also available as a Java package. There also exist dedicated analysis packages for pathway enrichment such as the BioPAX-Parser Java package (Agapito et al., 2020) and several tools solving the conversion of BioPAX representations into modeling formalisms such as the BioASF Java package (Haydarlou et al., 2016). Overall, however, there is no Python library implementing the BioPAX object model, and enabling the manipulation and analysis of BioPAX models.

## Summary

We present PyBioPAX, a Python software package to process and manipulate BioPAX models. PyBioPAX implements the BioPAX Level 3 object model as a set of Python classes, and implements a BioPAX OWL processor to deserialize BioPAX content from OWL files or strings into these objects. Once a BioPAX model and all its linked elements are deserialized into Python objects, they can be traversed and modified in memory. PyBioPAX supports serialization of BioPAX models into OWL/XML files compatible with other tools in the BioPAX ecosystem.

PyBioPAX implements the BioPAX OWL semantics where object attributes can be subtyped (e.g., “display name” is a subtype of “name”) using Python property attributes and getter/setter functions. It also supports exposing “inverse links” between objects; for example, a BioPAX Xref object, which represents a cross-reference, exposes a list of xref_of links back to the objects of which it is a cross-reference. Again, the coherence of these links at the level of a BioPAX model is guaranteed through the use of Python property attributes. The inverse links contribute to the efficient traversal of BioPAX models by allowing to link from e.g., one participant of a reaction to the reaction itself and its other participants. To facilitate model traversal, PyBioPAX provides a module to iterate over linked objects that satisfy a path constraint string specification from a given starting object.

PyBioPAX also provides a client to the Pathway Commons web service (Rodchenkov et al., 2020) that makes three different graph query types available: paths-from-to, paths-between, and neighborhood to extract subsets of knowledge aggregated from structured sources in Pathway Commons (e.g., Reactome (Jassal et al., 2020)) as BioPAX models. PyBioPAX further provides web service clients for processing BioPAX content from other pathway databases including NetPath (Kandasamy et al., 2010), and multiple members of the BioCyc database collection (Karp et al., 2019).

## Case studies

In the following case studies, we demonstrate the role of PyBioPAX in qualitative and quantitative analyses driven by BioPAX models.

### Traversing Pathway Commons

We demonstrate using PyBioPAX to process the Pathway Commons version 12 (PC12) “detailed” model BioPAX OWL file, to traverse it, and then to extract several biologically motivated motifs corresponding to the following questions:

1. Which controllers of the catalyses of biochemical reactions require a co-factor?
2. Which controllers of the catalyses of biochemical reactions are in a phosphorylated state?
3. Which biochemical reactions constitute a simple phosphorylation event?
4. Which complexes contain a protein bound to one or more small molecules?
5. What are all the features (e.g., post-translational modifications, fragments) of a given protein?

Our implementations of these queries in the corresponding Jupyter notebook identified nearly 4M objects in PC12, 83 controllers that need co-factors, 1,283 controllers that are in a phosphorylated state, 15,332 simple phosphorylation reactions, 13,338 proteins bound to a single small molecule, and 184 proteins bound to two more small molecules.

Additionally, PyBioPAX enabled us to write queries to find superlative entities. For instance, we found that the protein with the most modifications was NOTCH1, with 38 modifications. We further found that the RNA transcript of KTN1 had the most interactions (947), and AR had the most interactions of any protein (106).

### Gene set enrichment on Reactome pathways

Expert-curated pathways have been used as a means of dimensionality reduction and interpretation of transcriptomics data. However, most prior methods are limited to using pre-defined pathway lists (e.g., (Emon et al., 2020) only includes KEGG pathways). Here, we demonstrate using PyBioPAX to implement a similar workflow that is generally applicable to any pathway definition originating from BioPAX content, represented as PyBioPAX models.

First, we obtained all human pathways as PyBioPAX models through PyBioPAX’s API for the Reactome web service. We then traversed each model to identify physical entities representing proteins, aggregate their cross-references, and ultimately construct a list of HGNC gene identifiers for each pathway. Second, we collected curated transcriptomics experiments from the CREEDS database (Wang et al., 2016) that list the differentially expressed (DE) genes resulting from select drug perturbations, gene knockouts, gene overexpressions, and diseases.

Finally, we used Fisher’s exact test in an all-by-all comparison of the lists of DE genes for each perturbation experiment against the lists of genes whose proteins are present in each pathway. From this matrix we identified anti-correlations between drug perturbation experiments and gene perturbation experiments via the Pearson correlation coefficient. For example, this highlighted a strong relationship between estradiol and GPER1, suggesting GPER1 activation as a mechanism of action for estradiol.

The corresponding Jupyter notebook can be found here.

## Availability and usage

PyBioPAX is available as a package on PyPI with the source code available at https://github.com/indralab/pybiopax and documentation available at https://pybiopax.readthedocs.io/. The repository also contains an interactive Jupyter notebook tutorial and notebooks for the two case studies described above.

In addition to our case studies, PyBioPAX has been integrated into INDRA (Gyori et al., 2017) and serves as the primary entry point for processing BioPAX content into INDRA Statements through the traversal of a BioPAX model. It has also been used in (Weber et al., 2021) to process BioPAX content from Reactome into a node-edge graph used to train a machine-learning model used to improve natural language processing.
